# Optimized electroporation of CRISPR-Cas9/gRNA ribonucleoprotein complex for selection-free homologous recombination in human pluripotent stem cells

**DOI:** 10.1016/j.xpro.2021.100965

**Published:** 2021-11-16

**Authors:** Huaigeng Xu, Yuto Kita, Uikyu Bang, Peter Gee, Akitsu Hotta

**Affiliations:** 1 Department of Urology, University of California, San Francisco, San Francisco, CA 94143, USA; 2 Center for iPS cell Research and Application, Kyoto University, Kyoto 606-8507, Japan; 3 MaxCyte Inc., Gaithersburg, USA

**Keywords:** Cell isolation, CRISPR, Genetics, Molecular Biology, Stem Cells

## Abstract

Selection-free, scarless genome editing in human pluripotent stem cells (PSCs) by utilizing ribonucleoprotein (RNP) of CRISPR-Cas9 is a useful tool for a variety of applications. However, the process can be hampered by time-consuming subcloning steps and inefficient delivery of the RNP complex and ssDNA template. Here, we describe the optimized protocol to introduce a single nucleotide change or a loxP site insertion in feeder-free, xeno-free iPSCs by utilizing MaxCyte and 4D-Nucleofector electroporators.

For complete details on the use and execution of this protocol, please refer to Kagita et al. (2021) and Xu et al. (2019).

## Highlights

• A detailed protocol for efficient genome editing with Cas9/gRNA RNP electroporation
• Single-strand DNA template-mediated substitution of single nucleotide biallelically
• Targeted insertion of a short sequence, such as loxP site, without drug selection
• Establishment of knockin or knockout clones by seamless genotyping and subcloning

## Graphical abstract

**Figure:**
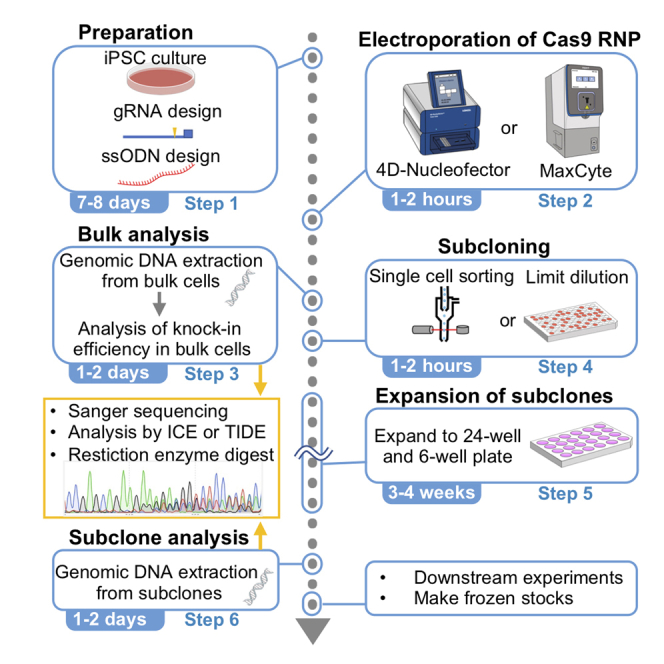

## Before you begin

The discovery of CRISPR-Cas9 has made tremendous progress in various research areas especially for genetic functions, disease modeling, and gene therapy (Dastidar et al., 2018; Ran et al., 2013; Soldner and Jaenisch, 2018; Zhong et al., 2020). This technology is widely applied to human PSCs, which have a great potential for regenerative medicine and cell therapies. With this in mind, the protocol utilizes feeder-free, xeno-free human PSCs cultured on recombinant Laminin-511 (iMatrix-511) with StemFit AK02N (or AK03N) media.

For successful genome editing, the method to introduce Cas9/gRNA complex into cells is critical. Ribonucleoprotein (RNP) form of delivery has several advantages over the DNA/RNA form (Gee et al., 2020; Kim et al., 2014; Paquet et al., 2016; Richardson et al., 2016; Zuris et al., 2015), since the RNP functions immediately, degrades quickly, and is relatively smaller than plasmid DNA or mRNA. Furthermore, the pre-formed Cas9/gRNA RNP complex can evade the inhibition by cellular mRNA (Kagita et al., 2021).

In addition to Cas9/gRNA, single-stranded oligodeoxynucleotides (ssODNs) must be available at the DNA repair site as a template for homology directed repair (HDR) pathway. In this protocol, we introduce Cas9/gRNA RNP complex and ssODN together into human PSCs by utilizing MaxCyte or 4D-Nucleofector electroporators, which were superior for inducing HDR.

Also, this protocol can be applied for single nucleotide editing for disease modeling and correcting mutations in PSCs, since more than half of disease-associated genetic changes are single nucleotide polymorphisms (Bamshad et al., 2011; Collins et al., 1997). Moreover, a 34 bp loxP sequence can be inserted for further genomic engineering by Cre recombination (Branda and Dymecki, 2004; Sauer and Henderson, 1988). Additionally, by simply omitting an ssODN, a small (∼10 nt) indel may be introduced for knocking out a target gene.

### Preparation of human iPSCs (feeder-free, Xeno-free condition)

**Timing: ∼8 days**

Prepare human iPSCs cultured in a well of 6-well plate with iMatrix-511 coating and StemFit AK02N (or AK03N) media as below. The original protocol developed by Dr. Nakagawa can be found in the below CiRA’s website.

(https://www.cira.kyoto-u.ac.jp/j/research/img/protocol/Ff-iPSC-culture_protocol_E_v140311.pdf)

**CRITICAL:** During culturing, iPSCs should be in the logarithmic growth-phase with minimal sign of differentiation or cell death. Passage at least two times after thawing before use. The condition and health of iPSCs are critical for this protocol because the HDR event only occurs in dividing cells at the S-G2 cell cycle phase.

***Alternatives:*** Although we have not tested alternative culture conditions in our hands, other feeder-free media (mTeSR, Essential 8, or DEF-CS) and coating materials (such as Matrigel, Vitronectin, or Geltrex) might be utilized. However, all the conditions, including seeding cell density and electroporation conditions, must be optimized for each culture system.

1. Plate coating.
  a. For coating an entire plate of 6-well plate, we add 50 μL (30–60 μL) of iMatrix into 12 mL PBS and mix them, and add 2 mL into each well of 6-well plate (final concentration: 0.25–0.5 μg/cm^2^). Required volume for different multi-well plates is summarized below.

**Table:** 

Plate	Amount of coating for each well
6-well plate	2 mL
12-well plate	1 mL
24-well plate	500 μL
96-well plate	100 μL
  b. Incubate at 37°C in a 5% CO_2_ incubator for 1–18 h.
    ***Note:*** Longer coating (such as overnight) is better for tight adhesion of iPSCs, and shorter coating (1 h) can be used for gentle dissociation of iPSCs before electroporation.
  c. Aspirate the coating buffer and add 2 mL of StemFit media + 2 μL of 10 mM Y-27632 (final 10 μM) into each well, and incubate in a CO_2_ incubator at 37°C for pre-warming the media until the dissociation of iPSCs is finished.

2. Passage of iPSCs from a well of 6-well plate
  a. Aspirate media of cultured iPSCs.
  b. Wash the cells by adding 1–2 mL of PBS, gently rock the plate, and aspirate PBS.
  c. Add 300 μL of 0.5 × TrypLE Select into iPSCs.
    ***Note:*** See “materials and equipment” section for making 0.5 × TrypLE Select. The half concentration of TrypLE Select solution provides better control and gentle detachment of iPSCs. Alternatively, accutase can be used as an animal product free cell dissociation reagent. Accutase contains collagenases, in addition to proteases, to facilitate detachment of cell types with rich extracellular matrix, such as iPSCs.
  d. Put the plate into a 37°C incubator and incubate for 10 min. Gently shaking the plate every 3–4 min is helpful for detaching the cells completely.
  e. Add 1 mL of StemFit media with 10 μM Y-27632 and gently pipet cells until iPSCs are dissociated completely.
  f. Count cells, and seed 1.0 × 10^4^–1.5 × 10^4^ cells into the iMatrix-coated 6-well plate which contains 2 mL of StemFit media with 10 μM Y-27632, as described in the Step c.
  g. Incubate cells in a CO_2_ incubator at 37°C for overnight.
  h. The next day, change media with 2 mL of StemFit media. If there are a lot of dead cells floating, keep adding Y-27632 to the media at the final 10 μM concentration.
  i. Change media every 2 days while culturing.
  j. iPSCs will be semi-confluent on day 6–8. Never let them become over-confluent. “Semi-confluent” means that iPSC colonies are less than 2 mm in diameter and there are still some spaces between iPSC colonies. The speed of growth depends on iPSC lines, so the timing of semi-confluency should be determined experimentally.
    ***Note:*** The addition of Y-27632 is important to improve the survival rate of iPSCs suspended in single cells (Watanabe et al., 2007). Keep adding Y-27632 during the culture in case there are many dead cells after seeding. If iPSCs do not reach semi-confluency after 8 days of seeding due to slow growth, passage the cells again with higher seeding cell density. As there are significant line-to-line variations among iPSC lines, we recommend switching the iPSC line if a certain cell line of iPSCs is difficult for culturing, electroporating, or genome editing.

### gRNA design for *Streptococcus pyogenes* CRISPR-Cas9 and preparation by *in vitro* transcription

**Timing: 2 days**

**Figure 1:**
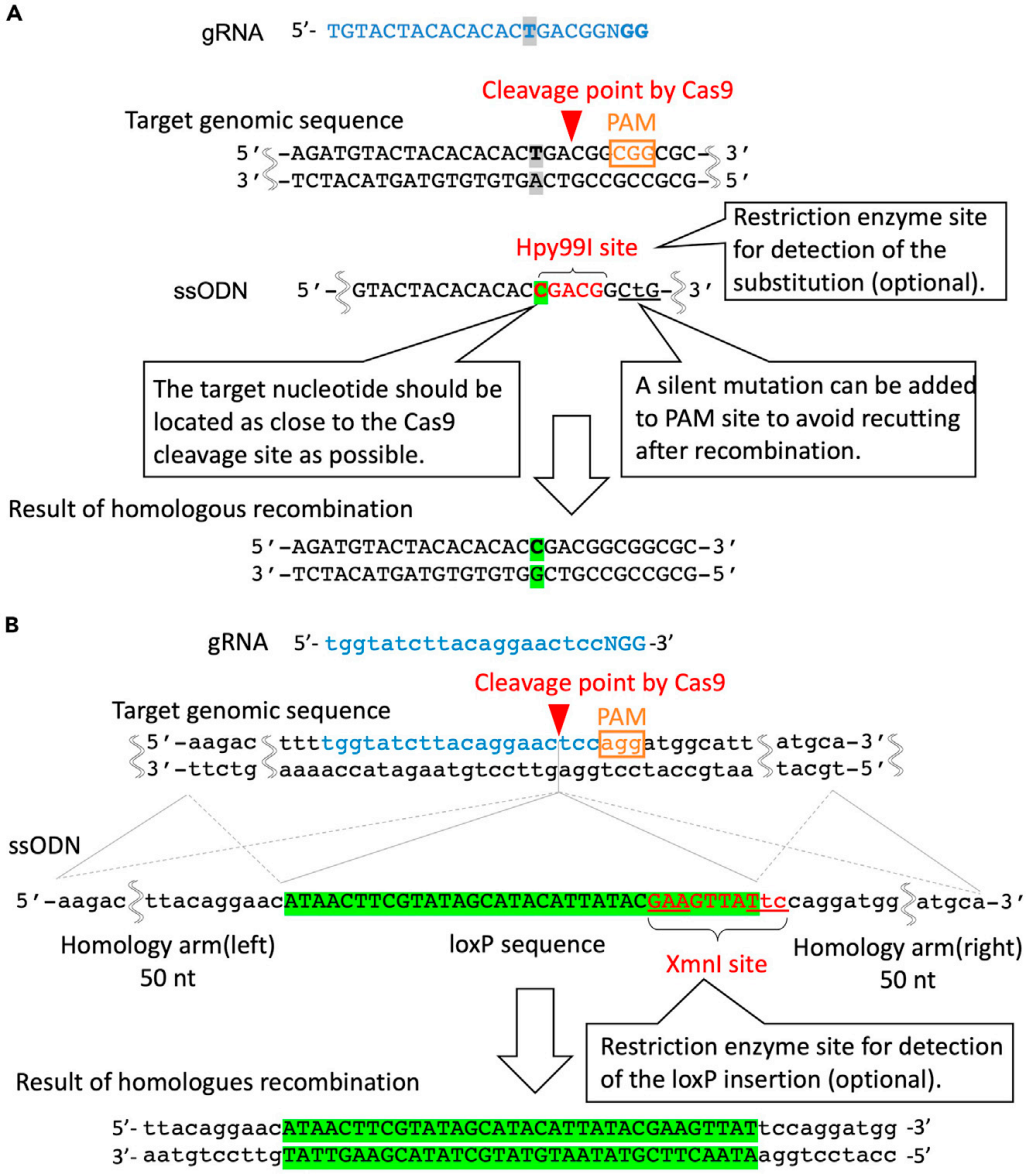
gRNA and ssODN design for single nucleotide substitution and loxP sequence knockin (A) An example of gRNA and ssODN design for single nucleotide alteration. First, the Cas9 cleavage site should be as close as the target SNP, ideally within 5 bp. ssODN template contains around 30–60 nt homology arms on both ends. In this case, the “T” base will be altered into “C” at 3-nt upstream (5′ side) from the Cas9 cleavage site. This conversion can be detected by the appearance of the Hpy99I restriction enzyme recognition site (5′-CGWCG-3′) as shown in red letters. Any other restriction enzyme site can be utilized, so long as the cleavage pattern by the restriction enzyme can be distinguished by gel electrophoresis. If it is difficult to design an appropriate restriction enzyme site after a single nucleotide alteration, the efficiency of substitution can be detected by Sanger sequencing. It is recommended to introduce a (silent) mutation at PAM or at the seed region of the target sequence to avoid recutting after recombination. (B) gRNA targeting site should be adjusted so that the Cas9 cleavage site is located as close to the loxP insertion site as possible. When two additional nucleotide sequence (TC) is added to the 3′ end of the loxP sequence, XmnI restriction enzyme site (5′-GAANN|NNTTC-3′) can be generated, which is later utilized to assess knock-in efficiency, as described in Figure 5.

3. Planning genome editing strategy and design of gRNA.
  a. Plan your genome editing strategy. Locate the nucleotide position and obtain the surrounding genomic sequence (i.e., from USCS genome browser, NCBI Nucleotide database, or IGV software).
  b. Search for a PAM sequence around the targeting site, ideally within a 10–20 bp window from the target site. SpCas9 utilize “5′-NGG-3′” PAM sequence (or “5′-CCN-3′” sequence for targeting reverse strand) and the expected cutting site is at 3 bp upstream (5′) from the PAM sequence (Figure 1). Choose several gRNAs if multiple candidate PAM sequences are found.
    ***Note:*** Several gRNA design tools such as CRISPick or Benchling are useful to identify the PAM sequence and gRNA site near the targeting site.
  c. Check the number of potential off-target sites by a web tool, such as CRISPRdirect or CRISPOR. Try to avoid gRNAs that target multiple potential off-target sites (less than 3 bp mismatch.) in the human genome. If multiple candidate gRNAs are found, choose gRNAs with fewer off-target sites. In addition, such mismatches are better located at the seed region (within 10 bp from PAM) since sequence recognition by gRNA is more precise at the seed region. Many gRNA design web tools provide a simple score that implement such mismatch preferences, so choose gRNAs with good score. If possible, we recommend designing several gRNAs for each target locus, as it is difficult to predict gRNAs’ activity and specificity until those are tested in cells.
  d. To prepare gRNA with *in vitro* transcription (IVT) reaction using T7 promoter, it is preferred that the target sequence starts from GG. This is because the first base of gRNA is the transcriptional start site for the T7 promoter, and gRNA transcription is more efficient if there are one or two Gs at the beginning of gRNA. Add two Gs in front of your gRNA sequence, or replace the first one or two nucleotides at the beginning of gRNA. Such additional Gs may decrease on-target efficiency and/or increase off-target cleavage risk. To avoid this complication associated with IVT reaction, order chemical synthesis of gRNA to completely match your gRNA sequence with the target site.
    ***Note:*** Design several gRNAs as described below to target the site of interest where you would like to alter the nucleic acid sequence or insert a small fragment such as the loxP site. When creating a conditional allele franked by two loxP sequence, make sure the insertion of the two loxP sites do not interrupt the genomic function, such as transcription and splicing. Stay away from such genomic motifs, if possible.
    ***Optional:*** If no proper PAM sequence is found within 10–20 bp window from the target site, consider utilization of engineered type II SpCas9 variants with unconstrained PAM sequence (i.e., SpCas9-VQR, VRQR, VRER, SpCas9-NG, xCas9, SpG, or SpRY), orthologs of SpCas9 (i.e., SaCas9 from S*taphylococcus aureus*), or other types of CRISPR (i.e., type V Cas12a/Cpf1 (Zetsche et al., 2015)). Note, only limited kinds of the engineered Cas9 variants are commercially available as RNP form (Cas12a RNP can be purchased from IDT). For this, try plasmid DNA transfection or protein purification.
    ***Optional:*** If the purpose of the experiment is gene knockout through NHEJ, there are multiple ways to prevent protein synthesis. The most major approach is to induce a small indel at the protein coding region (i.e. within exon) to disrupt the protein coding flame. In this case, be aware of the position of the indel if there are no alternative ATG start site(s) downstream or alternative isoforms that might skip the indel site. It is also better to make sure the expressed partial protein loses its functionality by deleting an important domain or residue.

4. Preparation of *in vitro* transcribed gRNA (IVT-gRNA)
  a. Design an oligo DNA as a template for IVT reaction. Insert your 20 bp-long gRNA target sequence (without PAM sequence, Green X letters) into the following primer. If your target sequence does not start from “G” or “GG”, then insert “**GG**” in front of the target sequence. If your target sequence starts from a “G”, then it is recommended to insert an additional “**G**” in front of the target sequence.
    T7-sgRNA forward primer (If the target sequence does not start from “G” or “GG”)
    5′-GAAATTAATACGACTCACTATA**GG**X_1_X_2_X_3_X_4_X_5_X_6_X_7_X_8_X_9_X_10_X_11_X_12_X_13_X_14_X_15_X_16_X_17_X_18_X_19_X_20_GTTTTAGAGCTAGAAATAGCAAG-3′
    T7-sgRNA forward primer (If the target sequence starts from a single “G”)
    5′-GAAATTAATACGACTCACTATA**G**X_1_X_2_X_3_X_4_X_5_X_6_X_7_X_8_X_9_X_10_X_11_X_12_X_13_X_14_X_15_X_16_X_17_X_18_X_19_X_20_GTTTTAGAGCTAGAAATAGCAAG-3′
    T7-sgRNA forward primer (If the target sequence starts from “GG”)
    5′-GAAATTAATACGACTCACTATAX_1_X_2_X_3_X_4_X_5_X_6_X_7_X_8_X_9_X_10_X_11_X_12_X_13_X_14_X_15_X_16_X_17_X_18_X_19_X_20_GTTTTAGAGCTAGAAATAGCAAG-3′
    ***Note:*** the gRNA target sequence in the forward primer needs to be put into 5′→3′ direction. DO NOT include PAM sequence (NGG) (i.e. “TGTACTACACACACTGACGG” is the sequence without PAM in Figure 1A).
  b. PCR amplify the gRNA template by the T7-fwd and T7-rev primers
    T7-rev primer: 5′-AAAGCACCGACTCGGTGCCACTTTTTCAAGTTGATAACGGACTAGCCTTATTTTAACTTGCTATTTCTAGCTCTAAAAC-3′
    PCR reaction

**Table:** 

PCR reagents	Final concentration	Amount
Ultrapure H_2_O		33 μL
10 × buffer	1 ×	5 μL
2 mM dNTPs Mix	0.3 mM	5 μL
25 mM MgSO_4_	1.5 mM	3 μL
T7-sgRNA forward primer (10 μM)	0.3 μM	1.5 μL
T7-sgRNA reverse primer (10 μM)	0.3 μM	1.5 μL
KOD-Plus-Neo (1.0 U/μL)	1.0 U/50 μL	1 μL
**Total**		**50** μL
    PCR cycling conditions

**Table:** 

Steps	Temperature	Time	Cycles
Initial Denaturation	98°C	30 s	1
Denaturation	98°C	10 s	30 cycles
Annealing	50°C	30 s	
Extension	68°C	15 s	
Final extension	72°C	10 min	1
Hold	4°C	∞
  c. Run the PCR product on a 2% agarose gel, and cut out the band with 121–123 bp in size.
  d. Extract the PCR band from the gel, and elute the DNA in 20–30 μL of RNase-free ultrapure water.
  e. Measure the DNA concentration by NanoDrop. The DNA concentration should be 20–50 ng/μL or higher.

5. *In vitro* transcription reaction
  a. Use MEGAshortscript™ T7 Transcription Kit for IVT reaction and mix the reagents as follow.

**Table:** 

In vitro transcription reagents	Final concentration	Amount
Ultrapure H_2_O		Up to 20 μL
10 × reaction buffer	1 ×	2 μL
75 mM GTP solution	7.5 mM	2 μL
75 mM ATP solution	7.5 mM	2 μL
75 mM CTP solution	7.5 mM	2 μL
75 mM UTP solution	7.5 mM	2 μL
T7 Enzyme Mix		2 μL
Template DNA	75–150 ng/20 μL	< 4 μL
**Total**		**20** μL

    ***Note:*** The template DNA volume is better with < 4 μL for minimizing carry-over of impurities from the PCR product.
  b. Incubate at 37°C for overnight (12–18 h)
  c. After the incubation, add 1 μL of TURBO DNase (attached in the kit) and incubate at 37°C for 15 min, for removing the template DNA.
  d. Extract gRNA by using the RNeasy MinElute Cleanup Kit. After the purification, we recommend eluting in relatively smaller volume of RNase-free ultrapure water (i.e., 30–40 μL) to obtain concentrated IVT-gRNAs. For better electroporation results, the concentration of the gRNA is better to be 1,000 ng/μL or greater.
  e. Aliquot IVT-gRNAs into 5–10 μL (5–10 μg) in each tube and store at −80°C. We recommend minimizing freeze-thaw cycles as much as possible. Thaw on ice right before use.
    ***Optional:*** If desired, gRNA can be synthesized by a company for nucleotide synthesis, such as IDT.

### Preparation of single-stranded oligodeoxynucleotide (ssODN) for a template of homologous recombination

**Timing: 2–3 days**

6. Design ssODN for homologous recombination (Figure 1).
  a. For replacing a single nucleotide in iPSCs, place the target nucleotide in the middle of ssODN and add 30–60-nt (typically, 50 nt) of homology arms in both sides. The position of the intended alteration site should be located at the 5′ end from the Cas9 cutting site (Paix et al., 2017). With this design, either sense or antisense strand ssODN can be utilized. An example of ssODN design for single nucleotide alteration is shown in Figure 1A. If a restriction enzyme site is designed to appear after ssODN-mediated HR, the frequency of ssODN-mediated HR can be estimated by restriction digestion. Also, a silent mutation should be added at the PAM site to avoid recutting after successful genome editing. To avoid introducing unnecessary amino acid mutations, refer to the codon table. If silent mutations cannot be added at the PAM site, introduce a silent mutation at the seed region of gRNA or near the Cas9 cleavage site. To insert a loxP site (5′- ATAACTTCGTATAGCATACATTATACGAAGTTAT-3′, 34 bp), the ssODN sequence can be designed as Figure 1B. Similar to above, the addition of two nucleotides “TC” would generate an XmnI restriction enzyme site (5′-GAANNNNTTC-3′), which can be used to assess the knock-in efficiency of the loxP sequence afterword. No silent mutation is needed in this case as insertion of the loxP sequence is sufficient to prevent recutting after recombination.
  b. Obtain ssODNs from a company for nucleotide synthesis (such as Fasmac, IDT, Hokkaido System Science) as a freeze-dried product. For most genome editing experiments, a basic purification method (i.e., reverse-phase cartridge purification) should be sufficient. If a higher efficiency is desired, select HPLC as a purification method.
  c. Dissolve the ssODN in ultrapure water at 10 μg/μL concentration. A high concentration of DNA is critical for efficient electroporation outcomes.

***Note:*** TE buffer may be used to reconstitute ssODN for 4D-nucleofector, but reconstitution in water is recommended for MaxCyte electroporation.

***Note:*** If homozygous targeting is necessary, choose your Cas9-cleavage site to be as close to the target base as possible. If heterozygous targeting is desired, and if current homozygous editing efficiency is too high, try designing your gRNA (Cas9 cleavage sight) slightly distal (5–15 bp) from the target base (Paquet et al., 2016).

This panel is re-used from Kagita et al. (2021).

## Key resources table

**Table:** 

REAGENT or RESOURCE	SOURCE	IDENTIFIER
**Chemicals, peptides, and recombinant proteins**		

iMatrix-511 silk (Laminin-511 E8)	Takara Bio	Cat#T311
Y-27632	Fujifilm Wako Pure Chemical Corporation	Cat#034-24024
StemFit® AK02N (or AK03N) media	Takara Bio	Cat#AK02N; AJ100
PBS (−)	Nacalai Tesque	Cat#14249-24
0.5 M EDTA	Nacalai Tesque	Cat#06894-14
Accutase	Sigma-Aldrich	Cat#A6964-500ML
TrypLE™ Select Enzyme (1×), no phenol red	Thermo Fisher Scientific	Cat#12563029
STEM-CELLBANKER® GMP grade	ZENOAQ	Cat#CB045
HyClone™ MaxCyte® Electroporation buffer	KIKO TECH	Cat#EPB-1
Recombinant SpCas9 protein	IDT, or Thermo Fisher Scientific	Cat#1081058, A36498
Lipofectamine Stem Transfection Reagent	Thermo Fisher Scientific	Cat#STEM00015
4-hydroxytamoxifen	Sigma-Aldrich	Cat#H7904
KOD-plus-Neo	Toyobo	Cat#KOD-401
PrimeSTAR® GXL DNA Polymerase	Takara bio	Cat#R050B

**Critical commercial assays**		

MEGAshortscript™ T7 Transcription Kit	Thermo Fisher Scientific	Cat#AM1354
RNeasy MinElute Cleanup Kit	QIAGEN	Cat#74204
Wizard® SV Gel and PCR Clean-Up System	Promega	Cat#A9281
P4 Primary Cell 4D-Nucleofector™ X Kit S (32 RCT)	Lonza	Cat#V4XP-4032

**Experimental models: Cell lines**		

1383D2 Human iPSCs, reprogrammed PBMC (LP_53, donor #40) isolated from a 36-years-old male donor by episomal vectors	Nakagawa et al. (2014)	From Dr. Nakagawa
DMD-iPSCs (CiRA00111 clone) from a male patient with Duchenne muscular dystrophy with exon 44 deletion (The sampling age is 1–9 years)	Li et al. (2015)	From Riken BRC https://cellbank.brc.riken.jp/cell_bank/CellInfo/?cellNo=HPS0383
Ff-XT28s05 iPSCs were generated from a healthy male donor homozygous for the 3rd frequent HLA haplotypes in Japan (A^∗^24:02, B^∗^07:02, C^∗^07:02, DRB1^∗^01:01)	CiRA Foundation, Xu et al. (2019)	https://www.cira-foundation.or.jp/e/project/genome-edited.html
404C2 Human iPSCs, reprogrammed skin fibroblasts (HDF1388) isolated from a 36-years-old female donor by episomal vectors.	Okita et al. (2011)	From Dr. Okita, RRID:CVCL_DP92
Oligonucleotides		
T7-DMDsgRNA1 forward primer: GAAA TTAATACGACTCACTATAgggtatcttacag gaactccGTTTTAGAGCTAGAAATA GCAAG	This Paper	N/A
T7-DMD-in55- g3-IVT forward primer: GAAA TTAATACGACTCACTATAggactttatagata tctcccaGTTTTAGAGCTAGAAATA GCAAG	This Paper	N/A
T7-DYSFgRNA2-IVT forward primer: GAAATT AATACGACTCACTATAggtactacacacact gacggGTTTTAGAGCTAGAAAT AGCAAG	This Paper	N/A
T7-sgRNA reverse primer: AAAGCACCGA CTCGGTGCCACTTTTTCAAGTTGATAAC GGACT AGCCTTATTTTAACTTGCTATTT CTAGCTCTAAAAC	This Paper	N/A
DMD1+loxPssODN (134-mer): AAGAC ATGGGGCTTCATTTTTGTTTTGCCTTT TTGGTATCTTACAGGAACATAACTTC GTATAGCATACATTATACGAAGTTAT TCCAGGATGGCATTGGGCAGCGGC AAACTGTTGTCAGAACATTGAATGCA	This Paper	N/A
DMD-in55- g3+loxP-ssODN (134-mer): TCA TTTGGAGGTAATTTGTTTGGAACAGTAT CAGACTTTATAGATATCTCATAACTTCGT ATAGCATACATTATACGAAGTTATCCATG GCTTGTGATAGAATATAAGGGCAATGC AAATGTAGAGTTTTTTGC	Kagita et al. (2021)	N/A
DMDexon55(45-55)check_dir5 primer: CCTCGGGTACACTGAAAGTTATGTG	This Paper	N/A
DMDexon45(45-55)check_rev5 primer: CACCACAGGCTTTAACTTCTGCCG	This Paper	N/A

**Software and algorithms**		

CRISPRdirect	Naito et al. (2015)	https://crispr.dbcls.jp
CRISPOR	Concordet and Haeussler (2018)	http://crispor.tefor.net
ICE	SYNTHEGO	https://www.synthego.com/products/bioinformatics/crispr-analysis
TIDE	Brinkman et al. (2014)	http://shinyapps.datacurators.nl/tide/
Primer3	Untergasser et al. (2012)	https://bioinfo.ut.ee/primer3-0.4.0/
Primer-BLAST	Ye et al. (2012)	https://www.ncbi.nlm.nih.gov/tools/primer-blast/

**Other**		

6-well plate	Greiner BIO-ONE	Cat#657160
12-well plate	Greiner BIO-ONE	Cat#665180
24-well plate	Greiner BIO-ONE	Cat#662160
96-well plate	Greiner BIO-ONE	Cat#655180
10 cm Cell Culture Dishes	Greiner BIO-ONE	Cat#664160-013
Falcon 5 mL Round Bottom Polystyrene Test Tube, with Cell Strainer Snap Cap	Corning	Cat#352235
MaxCyte® OC-100 cuvette	Kiko tech	Cat#SOC-1
MaxCyte ATx	Kiko Tech	Cat#E-ATx
4D-Nucelofector™ Core Unit	Lonza	Cat#AAF-1002B
4D-Nucelofector™ X Unit	Lonza	Cat#AAF-1002X
Applied Biosystems™ Veriti™ 96-Well Thermal Cycler	Applied Biosystems	N/A
Genetic Analyzer Trade-in for 3500 Series System	Thermo Fisher Scientific	N/A
NanoDrop™ 2000c Spectrophotometers	Thermo Fisher Scientific	Cat#ND-2000C
Agilent 2200 TapeStation	Agilent Technologies	Cat#G2965A
BD FACS Aria™ II cell sorter	Becton, Dickinson and company	N/A

## Materials and equipment

Make 0.5 TrypLE by adding an equal amount of 1 × TrypLE Select and 0.5 mM EDTA. An example is below.

**Table:** 0.5 × TrypLE solution

Solution	Final concentration	Amount
1 × TrypLE Select	0.5 ×	5 mL
0.5 mM EDTA	0.25 mM	5 mL
Total		10 mL

Store at 4°C for daily use. For long-term (>1 week) storage, store in −30°C.

***Alternatives:*** Accutase can be used instead of 0.5 × TrypLE solution.

## Step-by-step method details

### Electroporation of iPSCs by MaxCyte ATx electroporator (option 1)

**Timing: 1–2 h**

***Note:*** If you use 4D-nucleofector as transfection method, skip steps 1–4 and go to step 5.

MaxCyte electroporation of iPSCs enables higher efficiency and viability compared with other electroporation platforms under the conditions we have tested in the laboratory. We believe higher cell viability may enable better HDR efficiency when performing genome editing with CRISPR-Cas9 RNP and an ssODN template.

**Figure 2:**
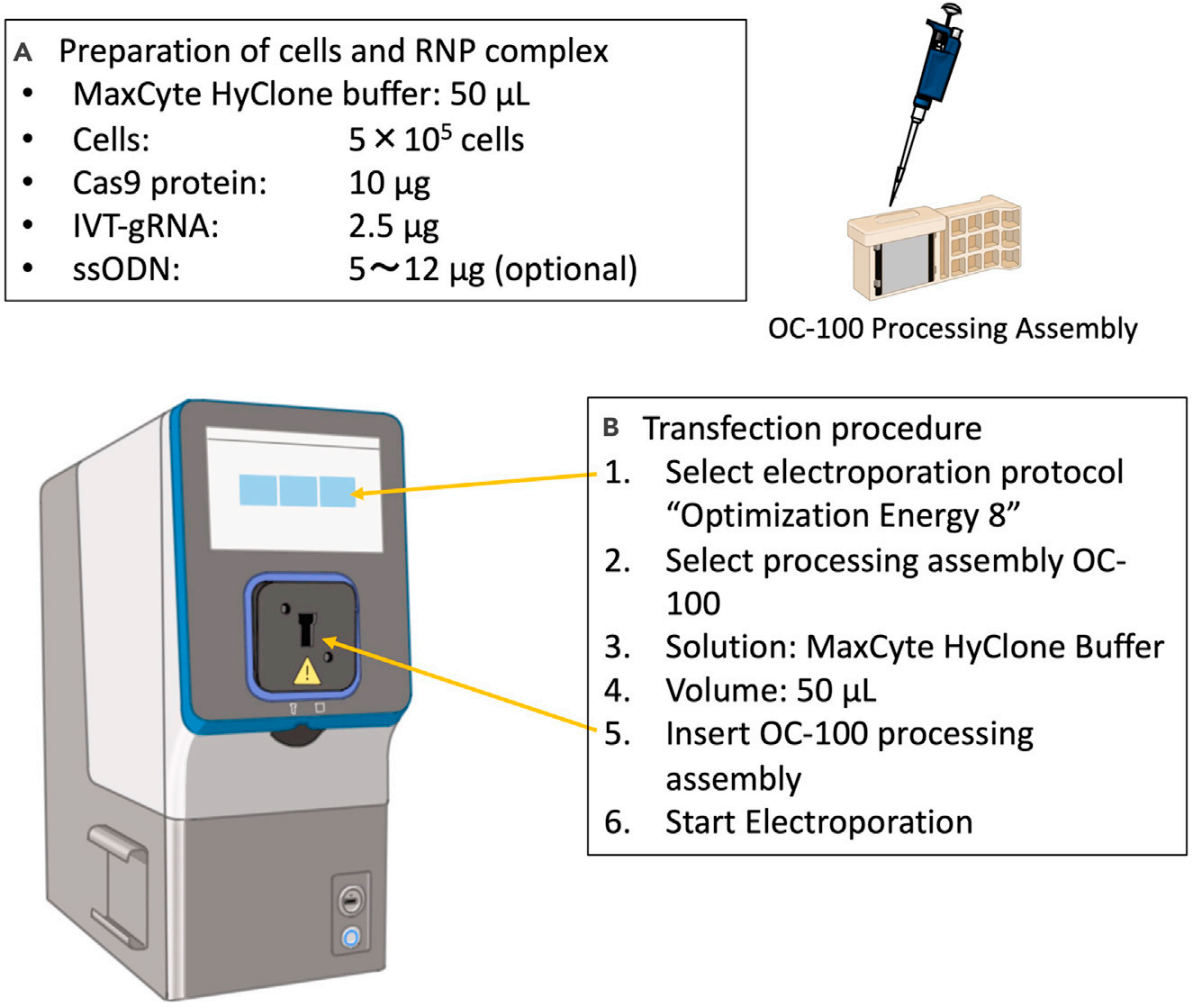
MaxCyte ATx mediated electroporation of RNP complex into iPSCs (A) Prepare a mixture of MaxCyte HyClone buffer with cells and RNP complex, and ssODN if applicable. Transfer 50 μL of the mixture into the well of OC-100 Processing Assembly chamber, after opening the lid. Then close the lid after the sample has been added to the OC-100. (B) Operation of the instrument. Select the desired electroporation protocol (Optimization Energy 8), and processing assembly (OC-100). Finally insert the OC-100 processing assembly and start electroporation.

1. Day-1 or Day-2: Passage iPSCs one or two days before electroporation at a high cell density
  a. For pre-coating of a 6-well plate with 0.25 μg/cm^2^ of iMatrix-511 (half of the usual concentration), prepare 2.5 μg of iMatrix-511 diluted in 2 mL of PBS. Then, coat wells at 37°C for at least 2 h in a humidified CO_2_ incubator. If more than one well is being coated, a master mix of the PBS and iMatrix-511 can be made in a 50 mL conical centrifuge tube and then added to each well.
  b. Wash iPSCs with 2 mL of PBS once and remove PBS.
  c. Add 0.5 mL of Accutase to the well.
    ***Alternatives:*** 0.5 × TrypLE solution can be used instead of Accutase. Accutase may be less toxic for iPSCs than 0.5 × TrypLE solution, especially at the timing of electroporation.
  d. Rock the plate every 3–4 min to ensure the Accutase covers the cells homogenously in the well.
  e. Incubate the plate at 37°C for 10 min. The cells should become rounded in the plate.
  f. Pipette the cells up and down in the Accutase solution with a 1000 μL pipette tip to detach the cells from the plate. Suspend the cells by pipetting while in the Accutase mixture to make sure the cells are dissociated well. Be careful not to pipette the cells too quickly to avoid shear stress, which will lower cell viability.
  g. Transfer the cell suspension to a new 1.5 mL microcentrifuge tube containing 0.9 mL of StemFit media.
  h. Centrifuge the cell suspension at 120 × *g* for 5 min.
  i. Aspirate the supernatant.
  j. Resuspend cells with 1 mL of StemFit media containing 10 μM Y-27632 by gentle pipetting.
  k. Count cells and check cell viability by trypan blue staining. The ideal cell viability is greater than 90%. If the cell viability is lower than 80%, it may indicate that the overall cell health is not optimal and more sensitive to electroporation damage.
  l. Plate 1.5 × 10^6^ cells of live iPSCs in the pre-coated iMatrix-511 plate in 2 mL of StemFit media containing 10 μM Y-27632. Incubate in a CO_2_ incubator at 37°C for one day.
    **CRITICAL:** The initial cell condition is critical. In our experience, passaging the iPSCs one or two days before electroporation has resulted in high HDR efficiencies. There may be some iPSC lines that are more fragile and may not respond well to passaging one day before electroporation. If this is the case, then the cells could be passaged two or three days before the above cell dissociation at a high cell density (i.e., 1.5–2.0 × 10^6^ cells/well in case 6 well plate).

2. Day 0: Preparation of the MaxCyte ATx electroporation instrument.
  a. Turn on the laptop PC connected to the MaxCyte ATx electroporation instrument at least 10 min before electroporation.
  b. After logging into the laptop PC, turn on the ATx instrument.
  c. Click the ATx shortcut icon on the Windows desktop.
  d. Select desired protocol for the experiment. Optimization 8 was the electroporation protocol established in our laboratory with several cell lines for efficient delivery of a variety of loading agents including DNA plasmids, mRNA, and CRISPR-Cas9 RNP.
  e. Select the OC-100 processing assembly from the processing assembly dropdown menu.

3. Day 0: Preparation of iPSCs for electroporation (Figure 2).
  a. 6-well plate with 0.25 μg/cm^2^ of iMatrix-511 (half of usual concentration) at 37°C for at least 1 h in a humidified CO_2_ incubator.
  b. Aspirate the coating buffer and add 2 mL of StemFit media containing 10 μM Y-27632 into each well, and incubate in a CO_2_ incubator at 37°C for pre-warming the media until finish the dissociation of iPSCs
  c. Perform the same procedures 1.b–i.
  d. Add 1 mL of MaxCyte HyClone Buffer to the cell pellet and pipet up and down gently to dissociate and wash the pellet.
  e. Count the cells and check for cell viability by trypan blue staining. The cell viability is suggested to be >80 %. Lower cell viability may indicate cells are not in optimal condition.
  f. Centrifuge at 120 × *g* for 5 min (at room temperature).
  g. Aspirate MaxCyte HyClone Buffer.
  h. Resuspend iPSC pellet in MaxCyte HyClone buffer at a cell density of 1 × 10^7^ cells/mL. Take into account the volume of the cell pellet itself when adding the MaxCyte HyClone buffer. For example, if there is the cell pellet with 1 × 10^6^ cells with around 10 μL of volume, add 90 μL of MaxCyte HyClone buffer to be the total 100 μL. For one electroporation condition, 50 μL of cell suspension is needed.
    ***Alternatives:*** Higher cell densities up to 1 × 10^8^ cells/mL can also be used without a loss in efficiency. Higher cell densities during electroporation can be considered in order to obtain a larger number of edited cells.
  i. Prepare CRISPR-Cas9 RNP mixture by mixing 10 μg of CRISPR-Cas9 protein with 2.5 μg of IVT gRNA and incubate at room temperature for at least 10 min in a 1.5 mL microcentrifuge tube.
    ***Note:*** The gRNA amount can be increased to 2–3 fold (5–7.5 μg). A higher ratio of gRNA to Cas9 protein ensures that free Cas9 is in a complex with gRNA, for improving knockout and knock-in efficiency.
  j. Then add 10 μg of ssODN into the Cas9/gRNA complex.
    **CRITICAL:** If this concentration of ssODN is causing low cell viability after electroporation, the amount of the ssODN can be titrated down in order to achieve better viability. The amount of ssODN can be optimized between 5 and 12 μg. Note, we found that high ssODN concentration tends to give a higher HDR rate, but is more toxic for cells.
  k. Add 50 μL of the iPSC cell suspension to the microcentrifuge tube containing the Cas9/gRNA complex and ssODN solution. Pipette up and down five times gently and avoid introducing air bubbles.
  l. Transfer the cell mixture to the OC-100 processing assembly slowly but steadily, only going to the first stop of the pipette in order to avoid the introduction of air bubbles. The OC-100 could be tapped gently to ensure the solution spreads out in the processing assembly. The mixture of single cells and CRISPR-Cas9/gRNA complex is not required to be incubated on ice before the electroporation step.
    **CRITICAL:** An air bubble in the cuvette may lead to an abnormal electric current. Make sure to avoid the addition of air bubbles. If found, make sure to remove it by using a 10 μL pipette. Also, make sure your cell suspension solution is evenly distributed (importantly, touching both electrodes on the side walls) in the well of the cuvette, so that an electric field will be evenly applied.
    **CRITICAL:** Do not leave the iPSCs sitting in the MaxCyte buffer for an extended period of time before electroporation. This may result in sensitizing the cells to more damage during electroporation.
    ***Optional:*** If the purpose of the experiment is gene knockout, omit the step ”j” and proceed to the next step.

4. Day 0: Electroporation of iPSCs
  a. Insert the OC-100 into the MaxCyte ATx electroporation port.
  b. Press “Start” on the instrument software.
  c. The first electroporation takes about 40 s for the machine to charge up. The actual electroporation is around 2–3 s.
  d. When you have more than 2 samples for electroporation, the electroporation from the 2^nd^ sample will take only 2–3 s as you do not have to wait for the charge
  e. After electroporation is completed, place the OC-100 into a 37°C humidified CO_2_ incubator for 20–30 min to allow for cell membrane recovery.
  f. After the recovery step, the cells can be transferred from the OC-100 to a well of 6-well plate with 2 mL of StemFit media containing 10 μM Y-27632 which is pre-coated with iMatrix-511 as described above.
  g. (Optional step) Rinse the OC-100 processing assembly with 50 μL of StemFit media in order to recover residual cells and add them to the culture vessel.
    ***Note:*** Cell viability can be affected by the seeding density. If you find significant cell death, try to use a smaller plate, such as 12-well plate for seeding.
  h. Incubate the cells in 37°C for culturing. Y-27632 can be removed after 1–2 days of transfection.
    ***Optional:*** if there are a lot of dead cells even after 2 days of transfection, keep adding Y-27632 in culturing media until cell recovery.
  i. After 3–5 days of transfection when cells become semi-confluent, dissociate cells. Expand a part of cells when it is necessary to maintain the cells, make a cell stock or extract genomic DNA.
    ***Optional:*** If there are enough cells, we recommend making a freeze stock of cells by STEM-CELLBANKER.
    **Pause point:** Once genomic DNA was extracted and frozen cell stocks were made, you may pause at this point.

### Electroporation of iPSCs by 4D-Nucleofector (option 2)

**Timing: 1–2 h**

***Note:*** If you use MaxCyte as transfection method, skip step 5–7 and go to step 8.

4D-nucleofector electroporation also enables high knockout and knock-in efficiency as the MaxCyte electroporator described above.

**Figure 3:**
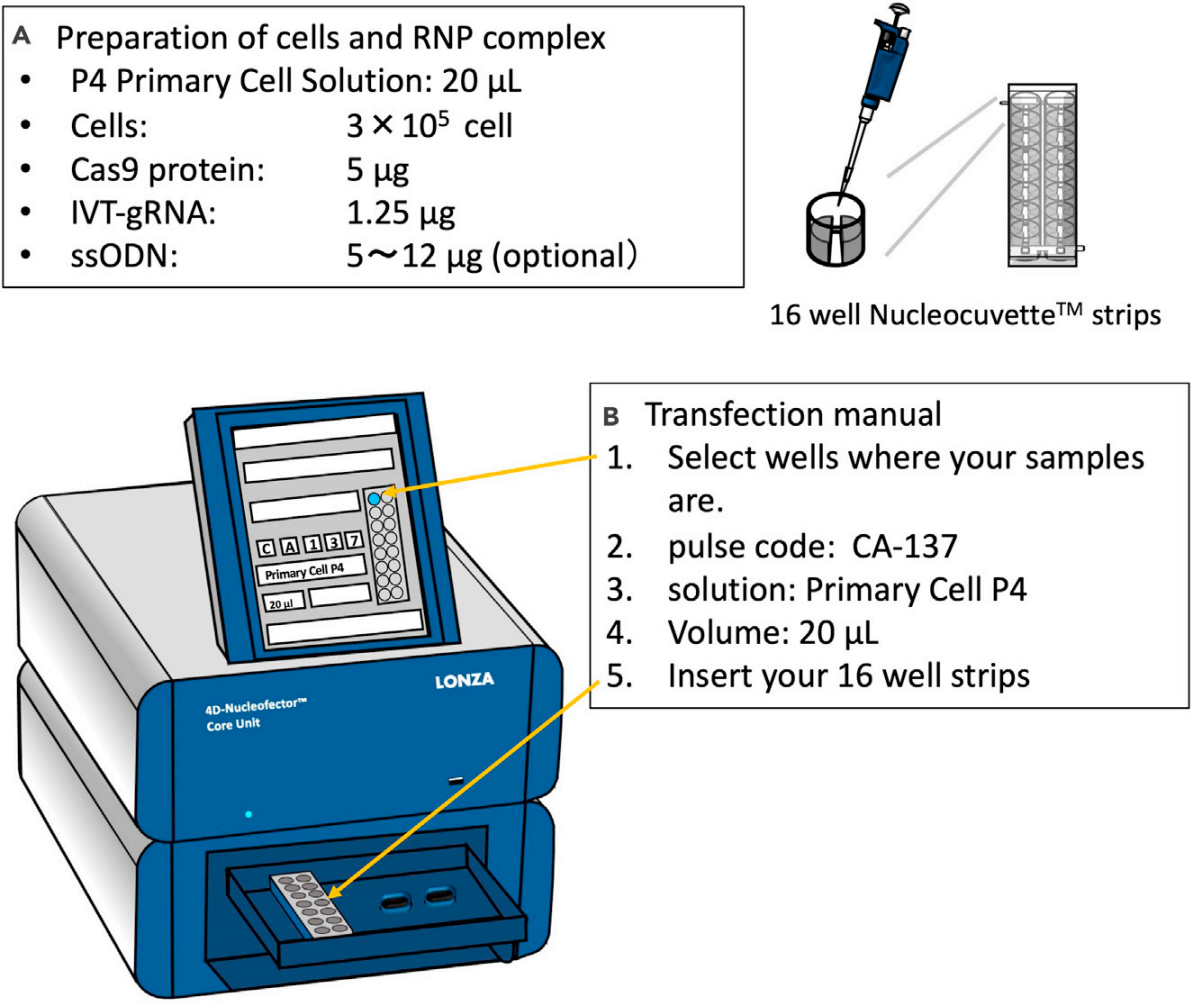
4D-Nucleofector mediated electroporation of RNP complex into iPSCs (A) Prepare mixture of P4 primary buffer with cells and RNP complex, and ssODN if applicable. Transfer 20 μL of the mixture into one well of 16 well strips. (B) Operation of the instrument. Select wells where your samples are, and then input pulse code (CA-137) and select solution (Primary Cell P4). Finally insert your 16 well strips and start electroporation.

5. Day-1 - 0: Plate coating.
  a. Prepare a 6-well plate where each well contains 2 mL of StemFit media containing 10 μM Y-27632 as described in MaxCyte part 3. a, b.

6. Day 0: Set up the 4D-nucleofector program for transfection (Figure 3)
  ***Note:*** This section can be done in parallel to “3. Preparation of iPSCs for electroporation”
  a. Turn on the 4D-nucleofector instrument.
  b. Chose X unit.
  c. Chose X-16 Strip (or X-single if you are using Single Nucleocuvette)
  d. Select the wells you are going to transfect.
  e. Set program as CA-137 and P4 primary buffer (or the buffer you are using).
    ***Optional:*** If necessary, other programs (CM-138, CM137, CM-150, DS-137, DS-138, and DS-130) also work for electroporating human iPSCs.
  f. Press “OK” for opening the tray port and proceed the cell preparation.

7. Day 0: Preparation of iPSCs for electroporation
  a. Perform the cell dissociation procedures as described in “Passage of iPSCs” section 2. b–d.
    ***Alternatives:*** For cell dissociation by Accutase, see the MaxCyte electroporation section.
  b. Perform cell counting using a hemocytometer and trypan blue.
  c. For one electroporation condition, add 3.0 × 10^5^ cells per condition in a 1.5 mL tube. If there are 5 conditions, multiple by 5 (i.e., 1.5 × 10^6^ cells).
  d. Centrifuge at 120 × *g* for 5 min (at room temperature).
  e. Aspirate supernatant.
  f. Add 20 μL of P4 Primary Cell Solution per condition into the cell pellet and gently suspend at room temperature. If you have 5 conditions, add 100 μL of P4 Primary Cell Solution.
    ***Note:*** P3 Primary Cell Solution can also be used, instead of P4 Primary Cell Solution.
  g. Prepare CRISPR-Cas9 RNP mixture by mixing 5 μg of CRISPR-Cas9 protein with 1.25 μg of IVT gRNA and incubate at room temperature in a 1.5 mL microcentrifuge tube.
    ***Note:*** 1.25 μg gRNA should be sufficient to 5 μg Cas9 in terms of molar ratio (roughly 1:1 molar ratio), however increasing gRNA to 2.5–3.75 μg may improve genome editing efficiency sometimes.
  h. Add 10 (or 6–12) μg of ssODN as a template for single nucleotide alteration or loxP insertion.
    **CRITICAL:** If this concentration of ssODN causes low cell viability after electroporation, the amount of the ssODN can be titrated down in order to achieve better viability. However, if the ssODN amount is too low, it may reduce knock-in efficiency.
  i. Transfer 20 μL of Cas9/gRNA and ssODN mixture into a well of Nucleocuvette Strip at room temperature. DO NOT make bubbles.
    **CRITICAL:** An air bubble in the cuvette causes an electric spark when high voltage is applied. Make sure to avoid air bubbles as possible. If you find one, make sure to remove it by using a P-10 pipette. Also, make sure your cell suspension solution is evenly distributed (especially, both sides of the electrode wall) in the well of the cuvette, so that electric field will be evenly applied.
  j. Set Nucleocuvette strip into 4D-Nucleofector.
  k. Press “Start” for starting electroporation.
  l. After electroporation, add 50–80 μL of StemFit media with Y-27632 into each well of Nucleocuvette Strip, pipet up and down several times gently, and transfer all cells into one well of the 6-well plate with 2 mL of StemFit media containing 10 μM Y-27632 which pre-coated with iMatrix-511 as described in the “Plate coating” section.
    ***Note:*** Cell viability can be affected by the seeding density. If you find significant cell death, try to use smaller plate, such as 12-well or 24-well plate for seeding.
  m. Incubate the cells at 37°C for culturing. Y-27632 can be removed after 1–2 days of transfection.
    ***Optional:*** if you see a lot of dead cells even after 2 days of transfection, you can keep adding Y-27632 in culturing media until cell recovery.
  n. After 4–7 days of transfection when cells become semi-confluent, dissociate cells. Expand a part of cells when it is necessary to maintain the cells, make a cell stock or extract genomic DNA.
    ***Optional:*** If there is a sufficient cell number, we recommend to make a freeze stock of cells by STEM-CELLBANKER.
    ***Optional:*** If the purpose of the experiment is gene knockout, omit the step “h” and proceed to the next step.
    **Pause point:** Once genomic DNA was extracted and frozen cell stocks were made, you may pause at this point.

### Assessment of genome editing efficiency in bulk iPSCs by Sanger sequencing

**Timing: 1–2 day(s)**

Assess overall genome editing efficiency in the electroporated iPSCs. This is an important step to validate your genome editing procedure worked or not, and to estimate how many subclones will be necessary to genotype from the bulk cells.

8. Genomic extraction
  a. Extract genomic DNA from the cell pellet by using MonoFas gDNA Cultured Cells Extraction Kit VI. This will be used for analyzing knockout efficiency in the next section. As a negative control, make sure to prepare genomic DNA from non-transfected iPSCs as well.

9. PCR and sequencing
  a. Design a pair of PCR primers to amplify the target locus by using Primer-blast or Primer3 software. Preferred PCR band size is around 500–800 bp for Sanger sequencing, and 150–200 bp for gel electrophoresis analysis for loxP insertion. The gRNA target locus should be located in the middle part of the amplified region.
  b. Amplify the target locus from the genomic sample(s) by PCR. Make sure to amplify from the non-genome edited sample as a negative control.
    PCR Reaction:

**Table:** 

PCR reagents	Final concentration	Amount
Ultrapure H_2_O		Up to 50 μL
5 × PrimeSTAR GXL buffer	1 ×	10 μL
dNTPs Mix (2.5mM each)	0.2 mM	4 μL
Forward primer (10 μM)	0.3 μM	1.5 μL
Reverse primer (10 μM)	0.3 μM	1.5 μL
Genomic DNA	100–500 ng/50 μL	X μL
PrimeSTAR GXL DNA Polymerase	1.25 U/50 μL	1 μL
**Total**		**50** μL
    PCR cycling conditions

**Table:** 

Steps	Temperature	Time	Cycles
Initial Denaturation	98°C	30 s	1
Denaturation	98°C	10 s	35 cycles
Annealing	X°C	15 s	
Extension	68°C	1 min/kb	
Final extension	68°C	10 min	1
Hold	4°C	∞

    ***Note:*** Try a higher amount (0.5–2 μg) of template genomic DNA if PCR amplification didn’t happen.
  c. Run on a 2% agarose gel to confirm a specific amplification. If a loxP sequence is inserted, you may see an additional band with 34-bp larger size. If you see non-specific amplification, optimize the PCR condition (i.e., raise annealing temperature). If desired, you may perform gel extraction to exclude the non-specific bands.
  d. Send the PCR sample for Sanger sequencing service. (i.e., GENEWIZ)

**Figure 4:**
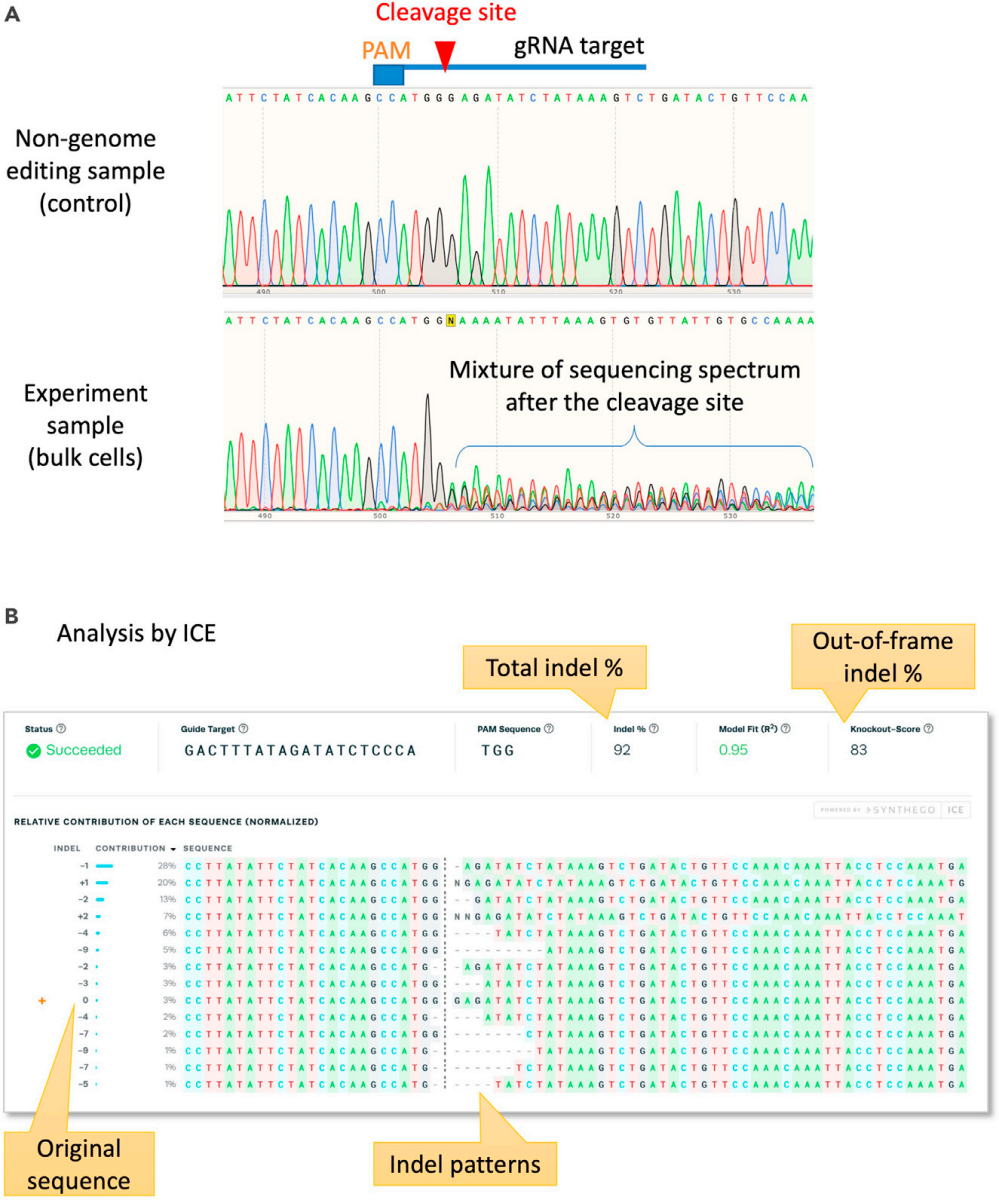
Indel pattern analysis by Sanger sequencing and ICE analysis (A) Cas9 and gRNA (DMD-in55-g3-gRNA: gactttatagatatctccca tgg) complex was electroporated into Ff-XT28s05 iPSCs by using 4D-nucleofector and P4 primary cell buffer. Sequencing results of the control sample (non-transfection) and experiment sample (transfected) were shown in upper panel. In the experiment sample, a mixture of spectrum can be observed after the Cas9 cleavage site. (B) Analysis result by ICE software. Indel percentage of the above Sanger sequencing data analyzed by ICE was 92 %, and knockout-score (exclude in-frame indels) was 83 %.
  c. Select the best condition (i.e., choice of gRNA or amount of ssODN) which shows the highest knock-in efficiency. The overall rate of expected genome editing outcomes is critical. If the efficiency is greater than 20–30%, proceed to the next subcloning step. If the efficiency is less than 10%, we recommend optimizing the experimental design or electroporation condition. Otherwise, it is necessary to screen hundreds of subclones for identifying a genome-edited clone."
  d. Expand the iPSCs that were confirmed for successful genome editing as described in the previous section. Make some frozen stocks as necessary.

10. Decomposition of Sanger sequence result by using ICE (Inference of CRISPR Edits) CRISPR Analysis tool.
  a. After you received Sangar sequencing result as “ab1” file, access to ICE web site and upload a control sample file and genome edited sample file. Alternatively, TIDE website can also be utilized.
  b. An example of ICE analysis for detecting NHEJ events is shown in Figure 4.

### Isolation of subclones of iPSCs and genotyping

**Timing: 3–4 weeks**

11. Day-7 - 0: Preparation of subcloning
  a. Harvest the semi-confluent iPSCs from the above section by 0.5 × TrypLE Select.
  b. Coat three 96-well plates with iMatrix-511 at least 1–24 h before cell seeding.
  c. If a cell sorter is available, proceed to single-cell sorting as described in step **12.** If not, proceed to limit dilution as described in step **13.**
12. Day 0: Sub-cloning by single cell sorting
  a. Perform the cell dissociation procedures as described in “Preparation of human iPSCs” section 2. b–d.
  b. Perform cell counting using a hemocytometer and trypan blue.
  c. Add 1.0–10.0 × 10^5^ of live cells in a 1.5 mL tube.
  d. Centrifuge at 120 × *g* for 5 min (at room temperature).
  e. Remove supernatant.
  f. Gently resuspend cells in 300 μL of FACS buffer (2% FBS in PBS). Transfer into a 5 mL flow tube with cell strainer to remove cell clumps.
  g. Use a cell sorter (i.e., BD FACS Aria II) to sort 1 cell/well of 96-well plate which prepared at part d. of “Day-7–0: Preparation of sub cloning”.
  h. Incubate the sorted 96-well plate at 37°C in a CO_2_ incubator.

13. Day 0: Sub-cloning by limit dilution **(Optional**)
  a. Perform the cell dissociation procedures as described in “Preparation of human iPSCs” section 2. b–d.
  b. Perform cell counting using a hemocytometer and trypan blue.
  c. Transfer 1.0 × 10^4^ of cells into a 15 mL tube with 10 mL of StemFit media containing 10 μM Y-27632.
  d. Further dilute the cell suspension by transferring 10 μL, 30 μL, and 100 μL into three 15 mL tubes with 10 mL of StemFit media and 10 μM Y-27632, so that the final concentration of the cells should be 10 cells, 30 cells, and 100 cells per 10 mL.
    ***Note:*** The number of single cell-derived subclones varies greatly from experiment to experiment, so we recommend to prepare three 96-well plates with different seeding densities (Suggested final seeding density for each plate could be 0.25 cells / 100 μL, 0.5 cells / 100 μL, and 1 cell / 100 μL, respectively). If the genome editing efficiency at the bulk stage was less than 10%–20%, preparation of more 96-well plates is recommended.
  e. Remove iMatrix coating buffer from the plate prepared in “Day-7–0: Preparation of sub cloning”.
  f. Add 100 μL of the cell suspension into one well of 96-well plate by using a multi-channel pipette. Make sure to suspend the cells frequently, to avoid uneven cell seeding.
  g. Incubate the plates at 37°C in a 5% CO_2_ incubator.
  h. The next day, observe each well of the 96-well plates under a microscope and mark the wells with single-cell seeding. A well with more than one cell should be discarded.

14. Day 2–14: After cell sorting or limit dilution (common step)
  a. After the single-cell seeding into a 96-well plate, there is a risk of cell loss during medium change. Instead of complete removal of media, add 50 μL of fresh StemFit media with 10 μM Y-27632 into a well of 96-well plate every 2 or 3 days.
  b. On day 6 or day 7, change media with StemFit (without Y-27632).
  c. Colonies will be visible at around day 7–10. Mark the wells with a single colony of iPS. If more than two colonies were observed, discard such wells.
  d. Add media for the wells with a single iPSC colony every 2 days (add 50 μL for 96-well plate)
  e. Around day 9-11, once the iPSC colony will become 100–300 μm in diameter, passage all the cells from one well of 96-well plate into a well of 24-well plate.
  f. Change media every 2 days until iPSCs become semi-confluent.
15. Day 14–21: After passaging cells from a single-cell derived colony
  a. When passaged cells become semi-confluent, extract genomic DNA from half of the cells by using MonoFas gDNA Cultured Cells Extraction Kit VI or a similar genome DNA extraction kit (i.e., Direct PCR). As the starting cell number is low, elute genomic DNA in a smaller volume (i.e., 40 μL) of elution buffer. Make a vial of freeze stock from the other half the cells using STEM-CELLBANKER.
  b. Repeat step a. for all single clones.
  c. When the number of samples has accumulated to some extent, perform PCR amplification of the target region and Sanger sequencing as described in the “assessment of genome editing efficiency in bulk iPSCs by Sanger sequencing” section. Repeat this step for all the isolated subclones or until desired subclones are identified.
  d. Choose the subclones which contain the expected knockout or knock-in sequence. More than 3 subclones are recommended for further downstream analysis (i.e., check for protein expression, cellular phenotype, pluripotency test, differentiation capacity, and genomic integrity).

## Expected outcomes

We examined if it is possible to induce a large (>300 kb) deletion using Cre-loxP reaction without antibiotic selection in iPSCs. As a proof-of-concept, we thought to introduce a 342 kb deletion between exon 45 and 55 of the dystrophin gene. We performed targeted insertion of two loxP sites by two rounds of ssODN mediated HDR. To insert two loxP sequences around exon 45 and exon 55, we designed two gRNAs to target each site, respectively. The loxP insertion site is adjusted to the Cas9 cleavage site, which is located at 3 bp upstream of the PAM sequence as shown in Figure 1B. Donor template ssODN for loxP insertion contains a 34 bp loxP site on the center and 50 bp homology arms on each side, that are homologous to the target locus on the exon 45 or 55.

**Figure 5:**
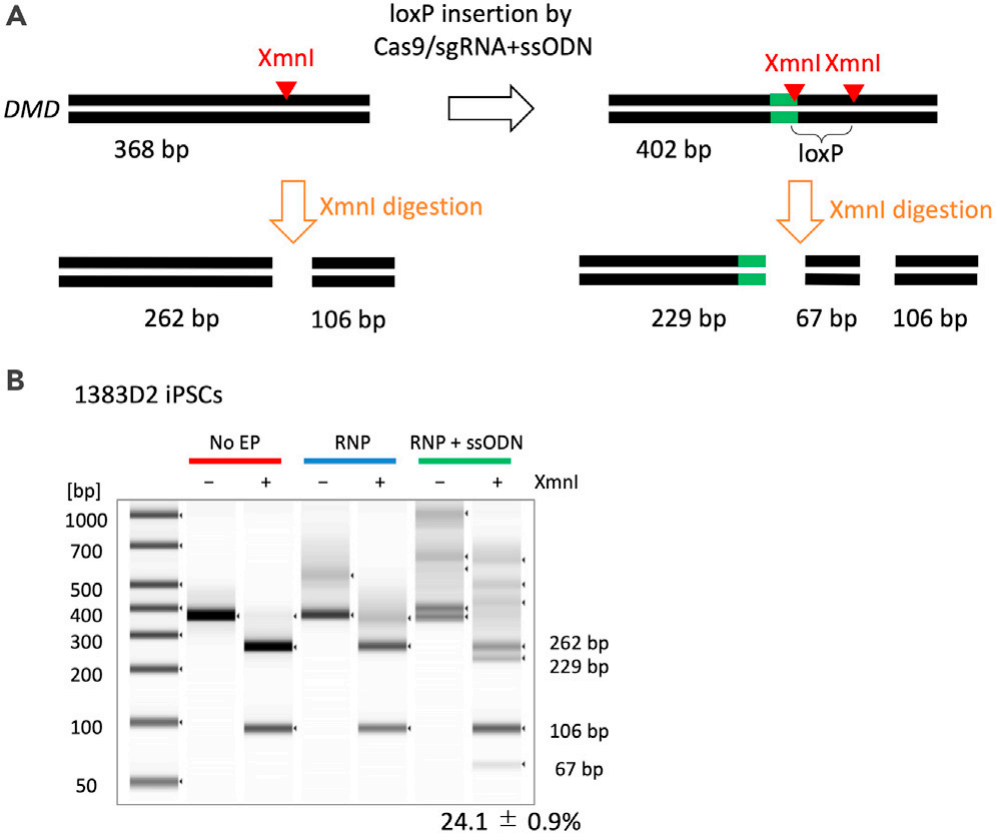
Insertion of loxP sequence using ssODN (A) Schematic to insert a loxP sequence at the DMD gene locus in 1383D2 iPSCs. Successful insertion of a loxP site generates an extra XmnI restriction enzyme site, which can be detected by DNA electrophoresis. (B) Cas9, gRNA, and ssODN were electroporated into 1383D2 iPSCs and genomic DNA was extracted for PCR amplification. TapeStation analysis of the PCR products with or without XmnI digestion. With the RNP and ssODN electroporation condition, extra bands with expected sizes (229 bp and 67 bp) were observed, suggesting the loxP sequence is inserted at the target site with around 24% of efficiency. This figure is adapted from Kagita et al. (2021).

We electroporated Cas9 protein and IVT-sgRNA (DMD-sgRNA1) complex together with the ssODN template (DMD1 + loxP-ssODN) into male iPSCs (1383D2 line) using MaxCyte with Optimization Energy 8. The first loxP knock-in was confirmed in bulk electroporated cells by genomic DNA PCR and subsequent restriction enzyme digestion by XmnI (Figure 5A). The insertion efficiency of loxP was more than 20% (Figure 5B) without antibiotic selection.

After confirmation of the 1st loxP insertion in bulk cells by restriction enzyme digest, we isolated subclones by single-cell cloning into a 96-well plate using BD FACS Aria cell sorter. Sanger sequencing of 20 subclones revealed that 8 subclones (40%) showed the intact loxP insertion, 8 subclones (40%) had unwanted indels, and 4 subclones (20%) were unedited. After finishing the first round of genome editing, we inserted the second loxP site around the exon 55 region by ssODN-mediated knock-in using the gRNA (DMD-in55-g3) and ssODN template (DMD-in55-g3+loxP-ssODN). Out of 14 subclones we analyzed by Sanger sequencing, 2 subclones showed successful insertion of the second loxP sequence.

To induce the excision of the 342 kb region flanked by the two loxP sites, 2 floxed clones were seeded into a 24-well plate (1.5–3.0 × 10^6^ cells/well) and 500 ng of tamoxifen-inducible Cre expressing plasmid (pCAG-IP-MerCreMer) were transfected with 1.5 μL of Lipofectamine Stem Transfection Reagent. 4–6 h after the transfection, 1 μM 4-hydroxytamoxifen was added to the media to induce nuclear translocation of Cre recombinase fused with a modified estrogen receptor (Mer). The excision of the floxed site (in this case, DMD exon 45–55 region) was confirmed by genomic DNA PCR in the bulk edited cells using “DMDexon55(45-55)check_dir5” and “DMDexon45(45–55)check_rev5” primers. In this PCR, the specific amplification appears only when the floxed site is excised. Then, subclones were isolated into several 96-well plates by a cell sorter and genotyped by the same PCR primers. We also performed Sanger sequencing of the PCR products and found that 4 subclones out of 11 clones showed excision of the DMD exon 45–55 region.

## Limitations

Our protocol is not necessarily applicable for all the genomic DNA sites because the CRISPR-Cas9 target site is restricted by PAM sequence specificity (NGG: in the case of SpCas9 gRNA). When gRNA activity is low, it is difficult to perform knock-in at a high frequency.

Since ssODN-mediated knock-in utilizes the HDR DNA repair pathway, the HDR pathway is required to be active (Paquet et al., 2016; Richardson et al., 2016). The HDR pathway is only active during the S phase and the G2 phase, hence the target cells should be actively proliferating (Gutschner et al., 2016; Howden et al., 2016; Mao et al., 2008) and our protocol is not applicable for non-proliferating cells.

Genome editing efficiency highly depends on the condition of the iPSC line to be utilized. Some iPSC lines might be difficult to handle due to slow growth, low survival, or spontaneous differentiation. In general, such iPSC lines are difficult to transfect and to edit by genome editing.

If there is a Single nucleotide polymorphism (SNP) in your gRNA target sequence, the SNP most likely interferes with the gRNA binding and subsequent Cas9 cleavage. Therefore, it is recommended to check the target sequence before designing gRNA.

## Troubleshooting

### Problem 1

No indel detected by Cas9/gRNA, without ssODN.

Step 10b at "assessment of genome editing efficiency in bulk iPSCs by Sanger sequencing" section.

### Potential solution

Use a positive control gRNA which has been shown to have cleavage activity in previous studies.

If your gRNA does not show cleavage activity despite the fact that the control gRNA does, check the sequence used in IVT and check the target sequence by Sanger sequencing.

Because Cas9/gRNA cleavage depends not only on the target sequence but also on the epigenetic state and many other factors. Therefore, no matter how carefully you design gRNA, some gRNAs have little cleavage activity. In that case, gRNA should be re-designed away from the original target site. To minimize the time burden, we recommend designing several gRNAs for each target site from the beginning.

In addition, previous studies have reported that gRNAs with extremely high or low GC content have low cleavage activity, so it is desirable that GC content of gRNA is within 40%–60% (Doench et al., 2014; Wang et al., 2014). It is necessary to avoid poly T (“TTTTT”) sequence from gRNA because it is a transcription termination signal when transcribed from polymerase III promoter.

If even a positive control gRNA does not show cleavage activity, re-check the condition of iPSCs and the RNP transduction step. Highly efficient genome editing is feasible only when iPSCs grow steadily in an undifferentiated state. In the RNP transduction step, it is important to mix Cas9 protein and gRNA well, because Cas9 protein is unstable until it binds to gRNA.

### Problem 2

Error in ICE or TIDE analysis and gRNA activity cannot be assessed.

Step 10b at "assessment of genome editing efficiency in bulk iPSCs by Sanger sequencing" section

### Potential solution

Although ICE or TIDE are useful because they analyze indels using Sanger sequence data, high quality Sanger electrogram data are necessary for analysis and the results are not reliable if the quality of the data is low (Brinkman et al., 2014). Nonspecific amplification in PCR causes a background noise. To avoid this, we recommend optimizing the PCR condition for specific amplification and performing agarose gel extraction of the PCR product.

If a sequence primer is designed close to the target site, it is not feasible to analyze the target sequence. Therefore, a sequence primer should be designed approximately 100 bp away from the target site. When analyzing a region that is difficult to sequence, such as a GC-rich region or a repeat region, re-consider a sequencing reaction or change the position of the primer.

### Problem 3

Although indels were actively induced by NHEJ, single nucleotide conversion/insertion via HDR was not detected.

Step 10b at "assessment of genome editing efficiency in bulk iPSCs by Sanger sequencing" section

### Potential solution

If the HDR mediated conversion/insertion does not occur even though double strand breaks are induced sufficiently, check the sequence of ssODN whether the homology arm matches the genomic DNA. Also,check the concentration of ssODN and the condition of the electroporation are appropriate. The optimal range of ssODN amount (typically 6–12 μg) varies depending on targeting locus and iPSC lines (Ishida et al., 2018). Optimize the amount of ssODN for each locus, each iPSC line.

It is important to use iPSCs in a logarithmic growth phase, because HDR occurs only in the late S and the G2 phase, and ssODN can reach the nucleus to serve as a template for HDR only when nuclear envelope disappears in the M phase (Howden et al., 2016; Mao et al., 2008).

The purity of synthesized ssODN affects electroporation efficiency and cell viability. To boost the HDR efficiency, it is worth trying high-purity purification methods such as HPLC.

HDR rate over NHEJ event is the locus- and cell type-dependent (Miyaoka et al., 2016). If you found it difficult to achieve HDR despite successful NHEJ events, consider changing the target site, or using a long, circular DNA template (i.e., plasmid DNA) with antibiotic resistance gene, to perform antibiotic selection of rare knock-in cells.

### Problem 4

After transfection, the cell condition is poor (such as low viability, differentiated, or do not grow).

Step 4i at the "electroporation of iPSCs by MaxCyte ATx electroporator (option 1)" section or Step 7n at the "electroporation of iPSCs by 4D-Nucleofector (option 2)" section

### Potential solution

It is inevitable that 20%–80% of cells die after transfection, but usually the cells recover from the damage within several days. If the cells do not grow at all or differentiate, it is possible that the transfection condition is too strong, therefore try a milder electric program or reduce ssODN amount. After electroporation, seeding into a smaller well might help as well to keep cell confluency.

### Problem 5

After sub-cloning, the cell condition is poor (such as low viability, differentiation, or loss of proliferation).

Steps at the "isolation of subclones of iPSCs and genotyping" section

### Potential solution

If the cells stop proliferation or get differentiated with the target gRNA sample, but not with the control gRNA, it is possible that your target is an essential gene of PSCs. If the problem is caused by the target gene after considering several gRNAs, it is necessary to use an inducible CRISPR system (Ishida et al., 2018) or create floxed allele to perform conditional knockout.

## Resource availability

### Lead contact

Further information and requests for resources and reagents should be directed to and will be fulfilled by the lead contact, Akitsu Hotta (akitsu.hotta@cira.kyoto-u.ac.jp).

### Materials availability

Genome edited iPSCs described in Kagita et al. (2021) will be available upon reasonable request, under MTA and ethical committee approval to use iPSCs derived from volunteers.

## Data Availability

The published article includes all datasets generated or analyzed during this study.
